# Mental health in Chilean family caregivers of people living with dementia: predictors of change in depression and anxiety symptoms over time

**DOI:** 10.1002/alz70858_102263

**Published:** 2025-12-25

**Authors:** Claudia Miranda‐Castillo, Andrea Slachevsky, Thamara Tapia‐Muñoz, Fabiola Gomez, Michel Madrid

**Affiliations:** ^1^ Universidad Andres Bello, Santiago, Chile; ^2^ Millenium Institute for Research in Depression and Personality, Santiago, Chile; ^3^ Millennium Institute for Care Research, Santiago, Chile; ^4^ Neuropsychology and Clinical Neuroscience Laboratory (LANNEC), Physiopathology Department ‐ ICBM, Neuroscience and East Neuroscience Departments, Faculty of Medicine, Universidad de Chile, Santiago, Chile; ^5^ Memory and Neuropsychiatric Clinic (CMYN), Neurology Service, Hospital del Salvador and Faculty of Medicine, Universidad de Chile, Santiago, Chile; ^6^ Geroscience Center for Brain Health and Metabolism (GERO), Santiago, Región Metropolitana de Santiago, Chile; ^7^ Servicio de Neurología, Departamento de Medicina, Clínica Alemana‐Universidad del Desarrollo, Santiago, Chile; ^8^ Universidad Andres Bello, Santiago, Santiago, Chile; ^9^ Universidad de Talca, Talca, Talca, Chile; ^10^ Millennium Institute for Care Research, Santiago, Santiago, Chile

## Abstract

**Background:**

The needs of people living with dementia (PLWD) vary throughout the course of the disease and this, in turn, may change the demands for care. These ongoing challenges can have a negative impact on caregivers’ mental health. This study aimed to determine the trajectories and predictors of depressive and anxious symptomatology in Chilean family caregivers of PLWD over two years.

**Methods:**

A telephone survey was conducted with 300 family caregivers of PLWD at baseline (T1) who responded to a survey about themselves, characteristics of the PLWD, and social factors. In the second wave, 208 carers participated (T2), and 155 in the third wave (T3). Latent growth curve and latent class growth mixture analyses were performed.

**Results:**

Caregivers’ mean age was 59.3 (SD=14.5) years and 78.9% were women. Fifty‐four percent were daughters and 34.8% were wives or partners of the PLWD. Regarding depressive and anxious symptomatology, it was found that both increased significantly over time (*p* <0.001). In addition, caregivers’ depressive symptomatology increased by 0.23 points for each point of increase in unmet needs of the PLWD (*p* = 0.04). Furthermore, anxious symptomatology increased by 0.82 points more in women compared to men (*p* = 0.01), and the greater the communicative impairment of the PLWD, anxious symptomatology increased by 0.13 points (*p* = 0.01).

**Conclusions:**

This is one of the few longitudinal studies of family caregivers of PLWD in Latin America, being the only one that has evaluated whether unmet needs of PLWD have a negative impact on caregivers’ mental health over time. It was found that the more unmet needs the PLWD have, the higher caregivers’ depressive symptomatology over time. At the same time, being a woman and caring for a PLWD with impaired communication increased caregivers’ anxious symptoms progressively. In consequence, it is essential to perform an ongoing screening for depressive and anxious symptomatology in caregivers, particularly in women; and, to offer comprehensive care to the PLWD‐caregiver dyad, since not addressing the needs of the former or treating mental health problems of the latter could result in a progressive deterioration on the quality of life of both.

